# A New Silicon Phase with Direct Band Gap and Novel Optoelectronic Properties

**DOI:** 10.1038/srep14342

**Published:** 2015-09-23

**Authors:** Yaguang Guo, Qian Wang, Yoshiyuki Kawazoe, Puru Jena

**Affiliations:** 1 Center for Applied Physics and Technology, College of Engineering, Peking University, Beijing 100871, China; 2 Key Laboratory of High Energy Density Physics Simulation, and IFSA Collaborative Innovation Center, Ministry of Education, Beijing 100871, China; 3 Department of Physics, Virginia Commonwealth University, Richmond, VA 23284, USA; 4 New Industry Creation Hatchery Center, Tohoku University, Sendai, 980-8577, Japan; 5 Kutateladze Institute of Thermophysics, SB RAS, Lavrentieva 1, Novosibirsk, 630090, Russia

## Abstract

Due to the compatibility with the well-developed Si-based semiconductor industry, there is considerable interest in developing silicon structures with direct energy band gaps for effective sunlight harvesting. In this paper, using silicon triangles as the building block, we propose a new silicon allotrope with a direct band gap of 0.61 eV, which is dynamically, thermally and mechanically stable. Symmetry group analysis further suggests that dipole transition at the direct band gap is allowed. In addition, this new allotrope displays large carrier mobility (~10^4^ *cm*/*V* · *s*) at room temperature and a low mass density (1.71 g/cm^3^), making it a promising material for optoelectronic applications.

Due to limited supply of fossil fuels and its adverse effect on the environment, one of the greatest challenges in this century is to find clean and sustainable energy sources. Of all the renewable energy sources available, solar energy provides the best solution^1^, but development of efficient photovoltaic materials to convert solar energy into electricity is essential. Silicon, because of its high stability and abundance, has been widely used as a photovoltaic material for solar cell devices. However, crystalline silicon does not absorb sunlight as efficiently as some other materials do. Its indirect band gap of 1.1 eV^2^ significantly limits the efficiency of solar cells as electron transition needs the assistance of phonons to change the momentum, thus, requiring a long optical pathway (thick film) for effective light absorption^2^,^3^. On the other hand, its direct band gap of 3.4 eV is too large^4^ and only allows high energy photons to be absorbed. Metastable silicon allotropes, such as R8, T12-Si, and lonsdaleite structures also exhibit indirect band gaps^5^,^6^,^7^ and the hotly-pursued silicene and BC8 phases show semimetal feature^5^,^8^. None of these materials are suitable for solar absorption. These limitations have led to a constant search for new silicon structures with small direct band gaps and desired optical properties.

Botti *et al.* studied several low-energy silicon allotropes with quasi-direct and dipole-allowed band gaps from 0.8 to 1.5 eV^9^. Similarly, Xiang *et al.* proposed a cubic Si_20_ phase with a quasi-direct band gap of 1.55 eV, while the dipole transition at direct band gap is allowed^2^. Although these structures exhibit better optical absorption than diamond silicon, their performance is not comparable with that of a direct band gap phase. Recently, using particle swarm optimization (PSO) algorithm, Wang *et al.* proposed six silicon structures. Among them, the oF16-, tP16-, mC12-, and tI16-Si show direct band gaps ranging from 0.81 to 1.25 eV^10^. Subsequently, using density functional theory (DFT) with generalized gradient approximation (GGA) for exchange-correlation potential at the PBE level, Lee *et al.* presented a series of direct band gap silicon crystals with band gaps ranging from 0.29 to 0.77 eV^11^. Even through these silicon structures can absorb sunlight at lower energies than other silicon allotropes and exhibit significantly enhanced optical absorption, there is a large room to enhance the conversion efficiency because photons with energy below the band gap cannot be absorbed^12^. This requires a small gap absorber to capture the low-energy photons. Note that photons in the visible and infrared spectra contain over 90% of solar energy.

In this paper we propose a three dimensional (3D) silicon structure built of Si triangles, h-Si_6_, whose dynamical, thermal and mechanical stability is confirmed by state-of-art theoretical calculations. Our results show that the h-Si_6_ phase has a direct band gap of 0.61 eV with good optical properties. The small effective mass and the large carrier mobility make h-Si_6_ a promising candidate for electronic device as well as solar absorber. The conversion efficiency of h-Si_6_ can be further enhanced by stacking it with other silicon allotropes having different band gaps^13^. We show that such an array can break the Shockley-Queisser limit^14^.

## Results and Discussion

**Figure 1:**
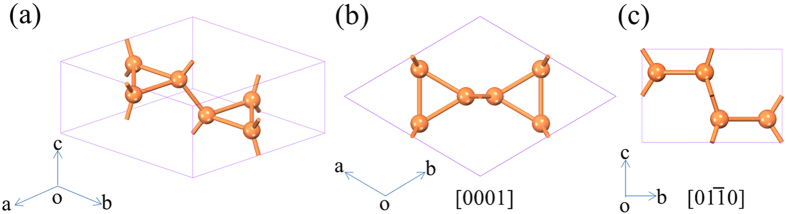
Optimized crystal structure of h-Si_6_. (**a**) Perspective view, (**b**) view from the [0001] direction, and (**c**) view from the 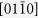 direction.

It has been demonstrated that the electronic structure of silicon crystals can be modified by varying the distribution of dihedral-angles while keeping the covalent bonds between the silicon atoms intact^11^. Similarly, the improvement of silicon’s optical transition and solar absorption has been shown to be closely related to its triangular motifs^2^. Thus, we conceived an idea of building a new 3D silicon structure with a direct band gap by combining these two factors. To accomplish this we changed the arrangement of silicon atoms in a unit cell by using the triangular motifs as building block. We constructed several possible structures before we reached the one with a pair of *up-down* equilateral triangles in a unit cell, as shown in Fig. 1. For convenience of discussion, we term this structure as h-Si_6_, since the newly designed phase has a hexagonal primitive cell (space group *P*6_3_/*mmc*, No. 194) containing six silicon atoms. The optimized lattice parameters are *a* = *b* = 6.94 Å, and *c* = 3.90 Å. This structure features 4-fold coordination with two bonds of 2.37 Å in the triangular building block and the other two of 2.33 Å between the silicon atoms in the neighboring triangles. The bond lengths are very close to that of diamond silicon (2.35 Å), showing a similar bonding character. Because there are three different bond angles among the silicon atoms (60°, 113.8°, and 118.2°), the silicon atoms show a characteristic of imperfect *sp*^3^ hybridization.

**Figure 2:**
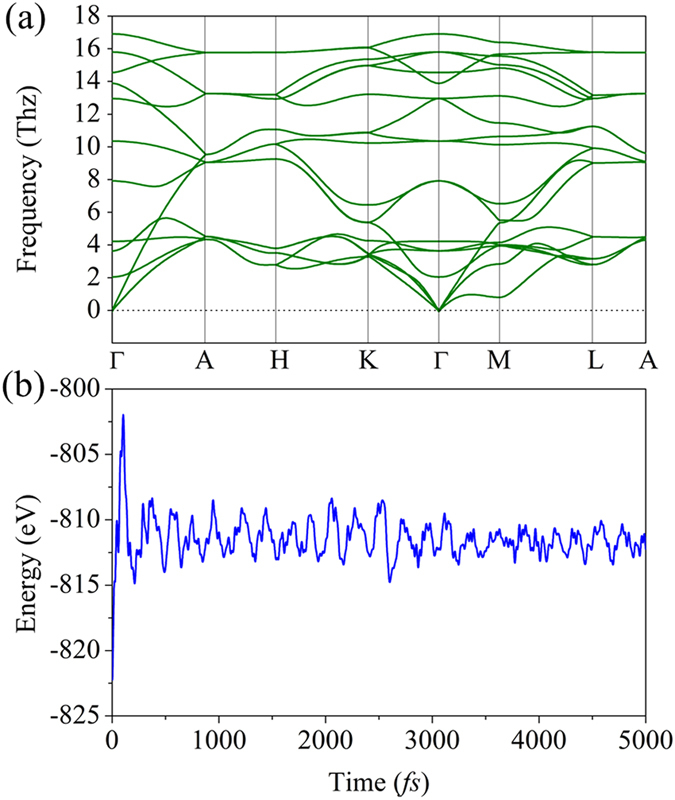
Structural stability of h-Si_6_. (**a**) Phonon dispersions and (**b**) energy fluctuation of h-Si_6_ with respect to time in MD simulations at 500 K.

To confirm the dynamical stability, phonon dispersion of h-Si_6_ was calculated by using the finite displacement method. The results are plotted in Fig. 2(a). We see that all the vibrational frequencies are positive in the first Brillouin zone, which clearly demonstrates that h-Si_6_ is dynamically stable and the structure belongs to a local minimum in the potential energy surface. We note that the highest frequency reaches 16.9 THz, which is slightly higher than that in diamond silicon (15.5 THz). We also checked its thermal stability from two different viewpoints. First, we calculated the total energy of h-Si_6_ and compared it with several other silicon allotropes. The degree of distortion is found to have direct impact on the energy level. The h-Si_6_ phase is 0.35 eV/atom higher in energy than that of the diamond silicon phase as no distortion occurs in the perfect *sp*^3^ configuration of the diamond structure. In h-Si_6_, the triangular three-membered rings lead to a larger distortion than the four- or five-membered rings in other direct band gap silicon crystals, such as oF16-, tP16-, mC12-, and tI16-Si phases. Therefore, h-Si_6_ is higher in energy by about 0.1 ~ 0.2 eV/atom than those previously reported direct band gap silicon structures^10^. However, the total energy of h-Si_6_ phase is comparable with that of the cubic Si_20_ phase, due to similar distortion of the Si tetrahedron^2^. Furthermore, h-Si_6_ is energetically more favorable than some experimentally identified silicon clusters, such as Si_4_, Si_6_, and Si_7_^15^. This result provides room for optimism that h-Si_6_ can be synthesized by some non-equilibrium growth processes as illustrated in previous studies^2^,^16^.

We then examined the thermal stability of h-Si_6_ by performing *ab initio* molecular dynamics simulations using canonical (NVT) ensemble. A large 3 × 3 × 3 supercell containing 162 atoms was used to reduce lattice constraint. The simulations were carried out with a Nosé thermostat^17^ at 500 K for 5 picoseconds with a time step of 1 femtosecond. The fluctuation of total energy with simulation time is plotted in Fig. 2(b). After 5000 steps, no obvious distortion in the structure appeared, and the average value of total energy remained nearly constant during the entire simulation. This implies that h-Si_6_ is thermally stable up to at least 500 K. The heat bath was further elevated to higher temperatures, and we found that h-Si_6_ can withstand temperatures as high as 1000 K. Thus, this new phase is separated by high energy barriers around its local minimum on the potential energy surface.

To study the mechanical strength of the h-Si_6_ phase, we have calculated its elastic response under external strain. In the linear elastic range, the elastic constant tensor forms a symmetric 6 × 6 matrix with 21 independent components. For a stable hexagonal structure, there only exist five independent elastic constants *C*_11_, *C*_33_, *C*_44_, *C*_12_, and *C*_13_, and they have to obey the condition^18^:
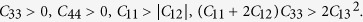


Using the strain-stress relationship^18^, we calculated the elastic constants of h-Si_6_. For comparison, similar calculations for the diamond silicon and cubic Si_20_ phase were also performed. All results are given in Table 1. Because the calculated elastic constants of this structure satisfy the above conditions, we conclude that that the h-Si_6_ structure is mechanically stable. The bulk and shear moduli were calculated using the Voigt-Reuss-Hill approximation^18^. The corresponding values for h-Si_6_ are 57.6 GPa and 32.8 GPa, respectively. Note that h-Si_6_ has a much smaller bulk and shear moduli than those of diamond silicon and cubic Si_20_ phase (see Table 1). In addition, it is also smaller than those of direct band gap hP12-Si and tP16-Si phases^10^ which are considered as the most porous structures in their proposed allotropes. To further compare the porosity with other 3D silicon structures, the mass density of h-Si_6_ was calculated. Besides being much lower than that of diamond silicon (2.34 g/cm^3^), h-Si_6_ has an even smaller density (1.71 g/cm^3^) than most previously reported bulk silicon allotropes^10^. This would help to reduce the material weight in practical applications.

**Figure 3:**
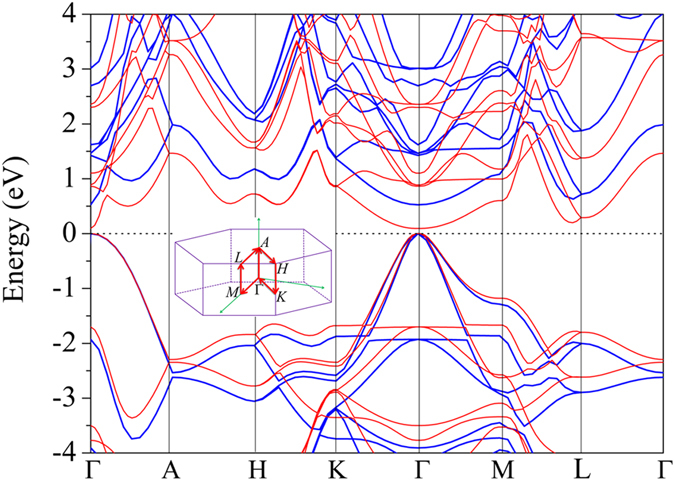
Electronic Properties. Calculated band structure of h-Si_6_ (red lines: GGA/PBE; blue lines: HSE06) with the Fermi level set to zero. The high symmetry *k*-point path in the Brillouin zone is shown in the inset.

For measuring optical absorption performance of the photovoltaic material, we have calculated the electronic structure of the h-Si_6_ phase because the conversion efficiency is closely related to the band gap^10^,^14^. The calculated band structure of h-Si_6_ throughout the hexagonal Brillouin zone is shown in Fig. 3. At the GGA/PBE level of density functional theory, the h-Si_6_ phase is predicted to be a direct band gap semiconductor with a small band gap of 0.14 eV, as both the valence band maximum (VBM) and conduction band minimum (CBM) are located at the Γ point. This value of 0.14 eV is less than half of the minimum band gap (0.29 eV) of previously reported direct band gap silicon crystals^11^. Because conventional DFT calculations are known to significantly underestimate band gap, we repeated the above calculations using the HSE06 functional. Although the band structure using the HSE06 functional is similar to that obtained at the PBE level, the band gap is increased to 0.61 eV. This value is much smaller than that of all the predicted direct band gap silicon allotropes at the HSE06 level. In addition, we note that the highest occupied state at the Γ point is doubly degenerate; this is because h-Si_6_ has hexagonal symmetry in the dihedral point group.

In a solar cell, when photoelectrons are produced, recombination of electrons and holes is not desired because this process decreases the solar conversion efficiency. Thus, large carrier mobility is needed^19^. Therefore, we investigated the charge transport properties of the h-Si_6_ phase using the deformation potential approximation^20^. The carrier mobility of a 3D solid can be written as 
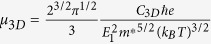
21, where *E*_1_ denotes the deformation potential, which is obtained by varying the lattice constant along the transport direction and studying the change of band energy under the lattice compression and strain. The effective mass m^*^ can be calculated by a quadratic polynomial fitting of the conduction and valence bands 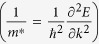 along several directions in wave vector space. The elastic modulus *C*_3D_ along different directions refers to the elastic constants given in Table 1; *C*_11_ represents the value along the *x*(*y*) directions while the *C*_33_ stands for the modulus in the *z*-direction. For an intuitive description, we rebuilt an orthorhombic crystal lattice along the three vertical directions 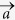, 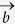, and 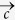 for h-Si_6_, as plotted in Supplementary Information Figure S1. The effective masses, deformation potential, and carrier mobility of electrons and holes along the three basis vector directions are listed in Table 2.

We begin our discussion by first looking at the carrier effective mass. Due to the intrinsic hexagonal structure, *m*_*x*_ = *m*_*y*_, showing an isotropic character in the *x-y* plane. For electrons, the effective mass along the *z* direction 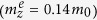 is smaller by an order of magnitude than that in the *x*(*y*) direction 
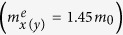
. This can be understood from the fact that the first conduction band has more significant dispersions along the Γ − *A* direction than that in the Γ − *M*(*K*) direction as shown in Fig. 3. For holes, we note that there are two valence bands near the Γ point, making the VBM a doubly degenerate state. So, there exist two kinds of holes, termed as light and heavy holes, respectively. Along the *x*(*y*) direction, the effective mass of heavy holes is −0.19*m*_0_, which is nearly twice that of the light holes (−0.10*m*_0_). Along the *z* direction, these two bands merge together, leading to an equivalent effective mass of −0.92*m*_0_ for both the light and heavy holes. Compared with the conventional semiconductor materials such as diamond silicon and GaN^22^, h-Si_6_ has a notable small effective mass feature, especially for the *x-y* plane holes and the *z*-axis electrons, making it a promising material for electronic devices.

The carrier mobility *μ* is strongly influenced by the effective mass as evidenced from the simple relationship *μ* = *eτ*/m^*^. In light of the small effective mass character, our predicted mobility of h-Si_6_ is impressively large at room temperature, around 10^3^ ~ 10^4^ *cm*^2^/*V* · *s*. In fact, this is much larger than that in the hotly pursued phosphorene^23^ and MoS_2_^24^. From a detailed standpoint, the electron mobility has the maximum value along the *z* direction, making it a high electron conductive channel. This phenomenon is a consequence of the very small effective mass, 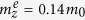, despite the fact that the deformation potential is extremely large, namely, 16.61 eV. For the hole mobility, on the contrary, the carrier in the *x*(*y*) direction is more mobile, since the effective masses of both light and heavy holes in the *x-y* plane are less than a fifth of that along the *z* direction. It is worth noting that the effective mass of light hole in the *x-y* plane is nearly equal to that of electrons along the *z* direction, but the former has about 3 ~ 4 times larger mobility than the latter one. This notable difference is due to the relatively small deformation potential for the light holes, which makes them less prone to acoustic phonon scattering than the electrons^25^,^26^. Our calculations show that the mobility for h-Si_6_ can reach as high as 10^4^ *cm*/*V* · *s* at room temperature, making it ideal for application as a photovoltaic material for solar energy conversion.

**Figure 4:**
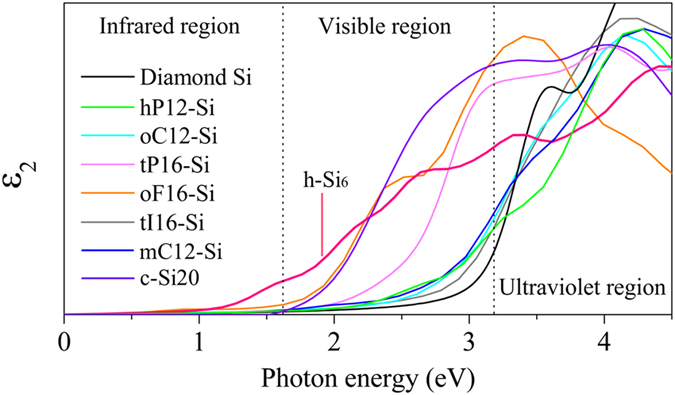
Optical properties. Imaginary part of dielectric function for h-Si_6_, diamond silicon, and other metastable silicon allotropes calculated at the HSE06 level.

The imaginary part of dielectric function of h-Si_6_ is calculated at the HSE06 level. For comparison, calculations were also carried out for diamond silicon and other metastable silicon phases studied previously^2^,^10^. The results are displayed in Fig. 4. According to the spectral range, we divided the spectrum into three parts, namely the infrared, visible, and ultraviolet regions, respectively. As mentioned above, due to the large direct band gap, diamond silicon can absorb photons mostly in the ultraviolet range. However, this part only occupies a small percentage of the sunlight, implying that plenty of energy is wasted. In addition, other metastable silicon allotropes can only absorb the photons with energy larger than about 1.5 eV, which is also not enough to make the best use of solar energy. For h-Si_6_ the absorption of low-energy photons starts from about 0.6 eV, which is very close to its direct band gap at the Γ point. Moreover, h-Si_6_ exhibits higher optical absorption than all the other silicon allotropes when the photon energy is below 2.2 eV. It also exhibits better optical absorption than diamond Si, hP12-Si, oC12-Si, tI16-Si, and mC12-Si from 2.2 to 3.4 eV. This implies that h-Si_6_ can be an ideal candidate for photovoltaic material. It was demonstrated that the maximum photoelectric conversion efficiency that can be achieved is 33.7% when the band gap is 1.34 eV for a single-gap photovoltaic device^12^. However, being tied to transition mechanism, the solar energy below this gap could not be really utilized, which inhibits the ability to further enhance the efficiency of solar cells. To overcome this limitation, a tandem solar cell system was proposed by stacking several homojunction cells together to form arrays, and it was found that a three- or four-stacked cell can bring the highest efficiency up to more than 60%, which requires a 0.5 ~ 0.6 eV band gap absorber to capture the low-energy photons^13^. The sketch of a tandem solar cell along with a detailed mathematical treatment for estimating the efficiency are given in the Supplementary Information. Thus, h-Si_6_ exactly satisfies the requirement, leading to potential application in photoactive layer, which would significantly increase the utilization of the sunlight.

**Figure 5:**
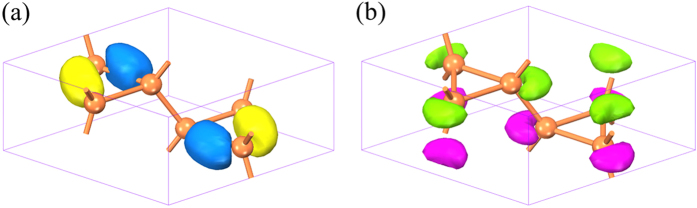
Calculated wave functions. (**a**,**b**) represent the VBM and CBM state at the Γ point of h-Si_6_, respectively.

To understand the reason why h-Si_6_ exhibits better optical absorption than previous Si allotropes, we examined the wave functions of the VBM and CBM plotted in Fig. 5. The VBM wave function is localized on the Si-Si bonds, while the CBM wave function is distributed around the Si atoms, indicating a bonding and antibonding state, respectively. The point group for the (0 0 0) *k* point is D_2h_, which has eight one-dimensional irreducible representations. It is found that the VBM belongs to *A*_*g*_ representation, whereas the CBM belongs to *B*_2*u*_ representation. In addition, for dipole transition, the dipole moment operator in D_2h_ group can be represented as the direct sum of B_1u_, B_2u_, and B_3u_. We then made a direct product of the three representations, i.e., 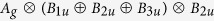. The result is 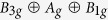, where the *A*_*g*_ representation is included, manifesting that the dipole transition at direct band gap is allowed. Interestingly, the wave functions of both the VBM and CBM are around the equilateral triangle units. Thus, it can be concluded that the optical transition is closely related to the silicon triangles, which is consistent with the similar mechanism observed in previous cubic Si_20_ structure^2^.

## Summary

To effectively harvest the sunlight in the whole wavelength range, it is a good strategy to use arrays consisting of Si structures with different direct band gaps. However, in this array the structure with a direct band gap of about 0.6 eV has been missing. In this study, we found this missing structure, the h-Si_6_ phase, by using silicon triangles as the building block. Using state-of-art theoretical calculations, we confirmed its thermal, dynamical, and mechanical stability. We further calculated its electronic band structure, optical properties and wave functions of the VBM and CBM. We also performed symmetry group analysis. We show that this new structure not only exhibits a good light absorption, but also exhibits high intrinsic mobility and low mass density. We hope that these features of the new Si allotrope will motivate experimental effort to synthesize the h-Si_6_ phase.

## Methods

Our studies are based on density functional theory (DFT) and the projector augmented wave (PAW) method^27^ as implemented in the Vienna *ab initio* Simulation Package (VASP)^28^. Plane waves with kinetic energy cutoff of 500 eV were used to expand the valence electron wave functions. The electronic exchange-correlation interaction was treated using Perdew-Burke-Ernzerhof functional (PBE) within generalized gradient approximation (GGA)^29^. To ensure an accurate determination of electronic and optical properties, calculations were repeated using the hybrid Heyd-Scuseria-Ernzerhof functional (HSE06)^30^,^31^. Full geometry optimizations were carried out by using the convergence thresholds of 10^−4^ eV and 10^−3^ eV/Å for total energy and force component, respectively. The Brillouin zone was represented with 9 × 9 × 16 Monkhorst-Pack special k-point mesh^32^. Thermal stability was studied using *ab initio* MD simulations with temperature controlled by Nosé heat bath scheme^17^. To study the dynamic stability, phonon calculations were performed using finite displacement method^33^ as implemented in the phonopy program^34^.

## Additional Information

**How to cite this article**: Guo, Y. *et al.* A New Silicon Phase with Direct Band Gap and Novel Optoelectronic Properties. *Sci. Rep.* **5**, 14342; doi: 10.1038/srep14342 (2015).

## Supplementary Material

Supplementary Information

## Figures and Tables

**Table 1:** Elastic constants and bulk and shear moduli of h-Si_6_, diamond silicon, and the cubic Si_20_ structure (GPa).

Structure	h-Si_6_	Diamond silicon	Cubic Si_20_
C_11_	83.0	160.7	147.3
C_33_	156.9		
C_44_	24.1	78.0	34.1
C_12_	34.3	63.9	46.3
C_13_	36.8		
B	57.6	96.2	80.0
G	32.8	64.5	40.2

**Table 2:** Effective mass (in *m*_0_), deformation potential (in eV), and carrier mobility (in 10^3^ *cm* ^2^/*V* · *s*) along the three stretching directions for electrons and holes of h-Si_6_ at 300 K.

**Carrier type**	***m***_***x***_	***m***_***y***_	***m***_***z***_	***E***_**1*****x***_	***E***_**1*****y***_	***E***_**1*****z***_	***μ***_***x***_	***μ***_***y***_	***μ***_***z***_
Electron	1.45	1.45	0.14	1.79	1.82	16.61	0.63	0.61	4.78
Light hole	−0.10	−0.10	−0.92	10.09	8.91	5.46	15.82	20.33	0.40
Heavy hole	−0.19	−0.19	−0.92	10.09	8.91	5.46	3.18	4.05	0.40
