# Supplementary material for: A New Silicon Phase with Direct Band Gap and Novel Optoelectronic Properties
Source: Sci Rep. 2015 Sep 23;5:14342. doi: 10.1038/srep14342 (PMC4585815; doi:10.1038/srep14342)
Supplement: Supplementary Information [file srep14342-s1.doc]

Supplementary Information

**A New Silicon Phase with Direct Band Gap and Novel Optoelectronic Properties**

Yaguang Guo^1,2^, Qian Wang^1,2,3,*^, Yoshiyuki. Kawazoe^4,5^, and Puru Jena^3^

^1^Center for Applied Physics and Technology, College of Engineering, Peking University, Beijing 100871, China

^2^Key Laboratory of High Energy Density Physics Simulation, and IFSA Collaborative Innovation Center, Ministry of Education, Beijing 100871, China

^3^Department of Physics, Virginia Commonwealth University, Richmond, VA 23284, USA

^4^New Industry Creation Hatchery Center, Tohoku University, Sendai, 980-8577, Japan

^5^Kutateladze Institute of Thermophysics, SB RAS, Lavrentieva 1, Novosibirsk, 630090, Russia

**Supplementary results**

1. **Structural parameters for the h-Si_6_ phase.**

**Table S1.** Geometrical parameters of h-Si6. (Lattice constants: a = b = 6.94 Å, c = 3.91 Å)

| Crystal | Space group | Wyckoff | *x* | *y* | *z* |
| --- | --- | --- | --- | --- | --- |
| h-Si_6_ | *P*6_3_/*mmc* | 6h | 0.552985 | 1.105970 | 0.250000 |

1. **Supplementary figures for carrier mobility.**

**Figure S1.** Schematic representation of the h-Si_6_ structure. The rhombus drawn with dashed lines represents the primitive cell. The super cell used for charge transport calculations (dashed rectangle) is also exhibited.

**Figure S2.** Linear fitting curves in the deformation potential. Band energy of the CBM as a function of lattice variation along three vector directions are plotted in (a), (b), and (c), respectively, while the (d), (e), and (f) represent the case of the VBM. The absolute values of different slopes stand for the different deformation potentials.

**3) Supplementary contents for the sketch of a tandem solar cell along with a detailed mathematical treatment for estimating the efficiency.**

According to the detailed balance principle used in study of the efficiency of tandem solar cell[^1^](#_ENREF_1), the limiting efficiency depends only on the various band gaps of solar absorbers. In Figure S3, we give a sketch of the architecture of a tandem structure using three homo-junction solar cells. Each of them absorbs a part of solar energy and a part of the electroluminescent spectrum emitted by other cells.

The mathematical treatment of the maximum efficiency is given below.

We consider the tandem structure of three homo-junctions solar cells as an example.

The *I-V* relationship of the *i*th cell is given by

$I_{i}(V_{i})=I_{0i}[\exp(qV_{i}/kT_{c})-1]-I_{1i}$.

The reverse saturation current $I_{0i}$ is determined by the radiative recombination between free holes and electrons[^2^](#_ENREF_2):

$I_{0i}=qF_{0i}=2qA\int_{E_{gi}/h}^{\infty} N(\nu,T_{c})d\nu$,

where A is the surface of a cell, $N(\nu,T_{c})=\frac{2\pi}{c^{2}}\frac{\nu^{2}}{\exp(h\nu/kT)-1}$ is the Planck black-body radiation flux, and $E_{gi}$ is the band gap of the *i*th cell.

The light-generated current is expressed as

$I_{1i}=q(F_{si}-F_{0i})$,

where *F_si_* is the photon flux incident to the *i*th cell.

For the first cell, apart from the solar illumination, it is illuminated by the light emitted by the second cell. Thus, *F_s_*_1_ is given by

$F_{s1}=A\int_{E_{g1}/h}^{\infty} N(\nu,T_{s})d\nu+A\exp(\frac{qV_{2}}{kT_{c}})\int_{E_{g1}/h}^{\infty} N(\nu,T_{c})d\nu$,

For the second cell, it is illuminated not only by the sun but also by the first and third cells. Therefore, *F_s_*_2_ is given by

$F_{s2}=A\int_{E_{g2}/h}^{E_{g1}/h} N(\nu,T_{s})d\nu+A\exp(\frac{qV_{1}}{kT_{c}})\int_{E_{g1}/h}^{\infty} N(\nu,T_{c})d\nu+A\exp(\frac{qV_{3}}{kT_{c}})\int_{E_{g2}/h}^{\infty} N(\nu,T_{c})d\nu$.

The third one will not be illuminated by the fourth cell as no more cell exists after it, so *F_s3_* is given by

$$F_{s3}=A\int_{E_{g3}/h}^{E_{g2}/h} N(\nu,T_{s})d\nu+A\exp(\frac{qV_{2}}{kT_{c}})\int_{E_{g2}/h}^{\infty} N(\nu,T_{c})d\nu$$

The total electrical power is defined as:

$P=-\sum_{i=1}^{n} V_{i}I_{i}$, where *V_i_* and *I_i_* are determined by the *I-V* curve of the *i*th cell.

Obviously, the maximum power of the *i*th cell can be obtained by$\partial P/\partial V_{i}=0$.

For the first cell, we have

$$\frac{\partial P}{\partial V_{1}}=-(I_{1}+V_{1}\frac{\partial I_{1}}{\partial V_{1}}+V_{2}\frac{\partial I_{2}}{\partial V_{1}})=0\text{ (1)}$$

In the above equation, (let $x_{i}=\frac{qV_{i}}{kT_{c}}$)

$$I_{1}=I_{01}(\exp x_{1}-1)-I_{11}=qF_{01}(\exp x_{1}-1)-q(F_{s1}-F_{01})\text{ (2)}$$

$$\frac{\partial I_{1}}{\partial V_{1}}=qF_{01}\frac{q}{kT_{c}}\exp x_{1}\text{ (3)}$$

$$\frac{\partial I_{2}}{\partial V_{1}}=-\frac{\partial I_{12}}{\partial V_{1}}=-q\frac{\partial F_{s2}}{\partial V_{1}}=-q\frac{F_{01}}{q}\frac{q}{kT_{c}}\exp x_{1}\text{ (4)}$$

Taking equations (2), (3), and (4) into equation (1), we obtain

$(1+x_{1})\exp x_{1}=\frac{F_{s1}}{F_{01}}+\frac{1}{2}x_{2}\exp x_{1}$.

Following the same procedure, we have

$$\left\{\begin{matrix} (1+x_{2})\exp x_{1}=\frac{F_{s1}}{F_{01}}+\frac{1}{2}x_{2}\exp x_{1}\text{ }(1st\text{ }\mathrm{cell}) \\ (1+x_{2})\exp x_{2}=\frac{F_{s2}}{F_{02}}+\frac{1}{2}x_{1}\exp x_{2}\frac{\int_{E_{g1/h}}^{\infty} N(\nu,T_{c})d\nu}{\int_{E_{g2/h}}^{\infty} N(\nu,T_{c})d\nu}+\frac{1}{2}x_{3}\exp x_{2}\text{ }(2nd\text{ }\mathrm{cell}) \\ (1+x_{3})\exp x_{3}=\frac{F_{s3}}{F_{03}}+\frac{1}{2}x_{2}\exp x_{3}\frac{\int_{E_{g2/h}}^{\infty} N(\nu,T_{c})d\nu}{\int_{E_{g3/h}}^{\infty} N(\nu,T_{c})d\nu}\text{ }(3rd\text{ }\mathrm{cell}) \end{matrix}\right.$$

We can get the maximum power (*P_m_*) by solving the above set of equations. *P_m_* is given by,

$$\begin{matrix} P_{m}=kT_{c}\exp x_{1}(x_{1}-\frac{1}{2}x_{2})F_{01}+kT_{c}x_{2}\exp x_{2}[(x_{2}-\frac{1}{2}x_{3})F_{02}-\frac{1}{2}x_{1}F_{01}] \\ \text{ }+kT_{c}x_{3}\exp x_{3}[x_{3}F_{03}-\frac{1}{2}x_{2}F_{02}] \end{matrix}$$

For the case of concentrated sunlight[^2^](#_ENREF_2), the maximum efficiency of the tandem structure composed of three homo-junctions is about 63%, with three optimal band gaps of 2.1, 1.2, and 0.6 eV.

Therefore, a tandem solar cell with more than three homo-junctions can reach the solar conversion efficiency over 60%.

**Figure S3.** Sketch of a tandem system with three homojunction solar cells ($E_{g1}>E_{g2}>E_{g3}$). The top cell converts high-energy photons with minimized thermalization loss and transmits the low-energy part of solar spectrum into the following cells.

**References**

1 De Vos, A. Detailed balance limit of the efficiency of tandem solar cells. *Journal of Physics D: Applied Physics* **13**, 839-846 (1980).

2 Shockley, W. & Queisser, H. J. Detailed balance limit of efficiency of p‐n junction solar cells. *Journal of applied physics* **32**, 510-519 (1961).
